# Development of antibody arrays for monoclonal antibody Higher Order Structure analysis

**DOI:** 10.3389/fphar.2013.00103

**Published:** 2013-08-21

**Authors:** Xing Wang, Qing Li, Michael Davies

**Affiliations:** Array Bridge Inc.St. Louis, MO, USA

**Keywords:** monoclonal antibody, Higher Order Structure, ELISA, biosimilars, comparability, conformational stability

## Abstract

Antibody arrays were developed to probe a monoclonal antibody's three-dimensional structure (3-D structure). Peptides with overlapping regions were designed to cover the whole mAb light chain and heavy chain, respectively, and used to generate polyclonal antibodies after the conjugation of the peptides to a carrier protein, KLH. It was shown that good peptide specificity was achieved from the antibodies generated. Using more than 30 different polyclonal antibodies to measure the surface epitope distribution, it was shown that the mAb antibody array can detect epitope exposure as low as 0.1% of defined mAb populations. This ELISA-based analysis of mAb epitope exposure can be considered as a measurement of “conformational impurity” in biologics development, similar to the analysis of other product-related impurities such as different forms of glycosylation, deamidation, and oxidation. This analysis of “conformational impurity” could provide valuable information on the mAb conformational comparability for biosimilar mAbs as well as novel mAbs, especially in the area of protein immunogenicity. Furthermore, stability studies indicated that there are several conformational “hot spots” in many mAbs tested, especially in the hinge region. This antibody array technology can be used for novel mAb Higher Order Structure (HOS) analysis during process and formulation development. Another important area of application is for biosimilar mAb development where the innovator molecule and biosimilar molecule could be compared based on their systemic “fingerprint” from the 30 plus antibodies.

## Introduction

Protein structure is the foundation of a protein's function. Numerous studies have demonstrated that protein three-dimensional structure (3-D structure) (Higher Order Structure or HOS) is critical for its biological function because the functions of all proteins rely on the precise spatial positioning of several functional groups with respect to each other (Kaiser and Kezdy, 1983). In biologics development, in addition to the importance of protein function, drug safety is also a major concern and protein immunogenicity is the focus of safety (Hermeling et al., 2004, 2005; Jiskoot et al., 2009). Throughout the history of biologics development, the immunogenicity of biologics has been reduced significantly. In the earlier days of biologics development, proteins of animal origin were used as therapeutics such as equine antisera, porcine/bovine insulin, this often resulted in significant immune response. Later on, human derived proteins were used for disease treatment such as human growth hormone and Factor VIII; however, the limitation of the human source limited the wide use of such biologics. Starting in the 1980s, with the development of biotechnology, many biologics could be produced efficiently in bacteria and other cultured cells, hastening the development of the biotechnology industry.

There are several consequences of immunogenicity in biologics. One of the major effects is a loss of efficacy because the antibodies generated against the biologics in turn will neutralize the biologics; more than a dozen cases of biologics have been reported showing this effect. A second consequence of anti-drug antibodies can be the enhancement of drug efficacy, sometimes this enhancement of efficacy could also cause a negative effect in the patient because it causes a biological imbalance for the homeostasis of the body. A third consequence of anti-drug antibodies can include the neutralization of endogenous proteins that share similar molecular structure to the biologic. Immunogenicity can also cause general immune effects such as allergy, anaphylaxis and serum sickness etc., and finally it is also possible that the anti-drug antibodies will not have any observed consequences. While the factors that influence the immunogenicity of biologics are many, they can be divided into two categories: (1) Product-related. This includes the sequence variation of the biologics, different impurity and contamination, product modification, and formulation. (2) Treatment related. This includes the application route, length of treatment, dose, and nature of the disease and the status of the patient.

In the past decade, great strides have been made in analytical technologies for the analysis of protein structure especially in the analysis of protein primary and secondary structure and post-translational modification such as glycosylation (which is closely related to a protein's function and immunogenicity potential). However, one area where more development is needed for an accurate and efficient structural analysis is determination of the 3-D structure (HOS) of biologics. In the recently published guidelines for biosimilar development, the US Food and Drug Administration (FDA) acknowledged that “a protein's 3-D structure is important but difficult to define using the current analytical technologies” (US Food and Drug Administration, 2012). There are several reasons why the analysis of protein HOS is such a challenge. On one side, it is the inherent complexity of the protein HOS. Many studies indicated that, different from a protein's primary and secondary structure, a protein's HOS could assume multiple conformations and the conformation is more dynamic than protein's secondary structure (James et al., 2003; Nobeli et al., 2009; Franco, 2011). This multiplicity makes some of the current technologies less informative in HOS analysis (Dyson and Wright, 1993). For example, CD (Circular Dichroism), Fluorescence and NMR have been used to analyze protein HOS, however, these techniques are not sensitive enough to detect many regional or minor but important conformational changes. Furthermore, the signal obtained by these technologies is an average of the whole population. They cannot reveal specific changes in a sub-population or regional changes in a molecule and it is this detailed information that is critical for an understanding of the function and potential immunogenicity of the biologics.

Given the limitations in the current analytical technologies for protein HOS analysis, efforts have been made to develop alternative technologies that could more accurately measure a protein's HOS. Hydrogen-Deuterium Exchange (HDX) is a technology that has been available for more than 20 years (Houde et al., 2009, 2010, 2011; Wei et al., 2012). It has gained momentum recently for the analysis of protein HOS due to improvements in mass spectrometry and software for data processing (Jacob and Engen, 2012; Pascal et al., 2012; Xiong et al., 2012). However, there are some limitations for HDX technology, one of the major challenges is its technological complexity. Extensive Mass Spectrometry expertise is needed to carry out the analysis and data processing. These factors limit its use as a routine assay in biologics process and formulation development or for comparability studies. Another limitation to HDX is assay accuracy. Because of the complexity of the process that involves proteolytic digestion, chromatography, and MS for protein identification, a highly reproducible result is often difficult to obtain. Therefore, a more sensitive and accurate technology is desirable in the area of protein HOS analysis. Recently, aptamers has been used to study therapeutic protein structural changes with some very interesting findings; however, more studies are needed to demonstrate the value of this novel technology in monoclonal antibody conformational analysis (Sominskaya et al., 1992).

In immunology, it is known that 5–6 amino acids are adequate to form an epitope for antibody generation; there are also reports of 3–4 amino acids that can define an epitope (Nestor, 2009; Zichel et al., 2012). The current study reports an antibody array-based approach to measure monoclonal antibody surface epitope distribution that will reflect the protein's HOS. First, peptides with overlapping regions were designed to cover the whole sequence of a protein. For a monoclonal antibody, a total of 34 different peptides were used to cover the mAb light chain and heavy chain, respectively. Second, all the peptides are conjugated to a carrier protein such as KLH (Keyhole Limpet Hemocyanin) to increase the peptide immunogenicity and retention time in the host. Another consideration in the development of the antibody is the length of the peptides. Previous studies indicated that peptides as short as 11 amino acids could form secondary structure such as α-helix (Dyson and Wright, 1991; Thomas et al., 2006; Maupetit et al., 2009). During the development of the antibody arrays, peptides with amino acid length between 25 and 30 were designed that, in theory, would allow the formation of some secondary structure such as α-helix and β-sheet. The intention was to generate antibodies that also react to some of the secondary structure features of the protein.

Since the principle of the antibody array technology is based on epitope recognition, it was expected that the signal obtained from this antibody-based measurement would measure structural features at the molecular level (Shon et al., 1991), and more importantly, because each peptide corresponds to a certain region of the protein, if the peptide-derived antibody demonstrates good specificity, this approach would allow the regional (local) assignment of the recognized change. This report is the summary of the development of multiple antibody arrays toward many best-selling monoclonal antibody therapeutics currently on the market and also one set of antibody arrays for novel mAb development. It was shown that sensitive and accurate information could be obtained using this antibody-based approach, and this information could be used for the development of novel therapeutic mAbs as well as biosimilar mAbs, case studies were presented in these two important application areas.

## Materials and methods

### Reagents

All the chemicals were purchased from Sigma-Aldrich (St. Louis, Missouri). 96-well microplates were purchased from Corning Co. (Corning, New York). Streptavidin-HRP conjugate and biotin labeling kits were obtained from Thermo Scientific (Rockford, Illinois).

### Antibodies and ELISA kits

All the antibodies and ELISA kits used in this study were products of Array Bridge Inc. (St. Louis, Missouri). Polyclonal antibodies against the peptides were produced in New Zealand White Rabbits. For the sandwich ELISA, antibodies against each region of the mAb molecule were coated on 96-well plates with each antibody coating a column in row B through G. In each column of the coated plates, the upper three wells (B, C, and D) were incubated with a reference mAb such as an innovator mAb (marketed mAb) in triplicate, and the lower three wells (E, F, and G) were incubated with a biosimilar mAb candidate in triplicate. A biotin-labeled rabbit anti-human IgG antibody was used to detect the mAb-peptide antibody complex, and streptavidin-HRP was used to detect the complex formed by anti-human IgG-mAb-peptide antibody. The signal strength of the sandwich ELISA depends on the relative epitope exposure of the mAb in each region. If there are more epitopes from the mAb that could be recognized by the peptide-derived antibody, a stronger signal will be produced and vice-versa.

### Sample treatment

For urea-denatured monoclonal antibodies, urea at a final concentration of 8 M was added to a 10 mg/ml mAb solution and incubated overnight at 4°C. The mAb solution was subsequently diluted to 5 μg/ml for ELISA analysis. For heat treatment, mAbs at 2 mg/ml in PBS were treated at indicated temperature overnight and then diluted to 5 μg/ml for analysis.

For the sandwich ELISA, six rows of anti-peptide antibodies were coated in 96-well plates from row B–G. For comparability studies, the first three rows (B, C, D) were used for reference mAb, typically the innovator mAb (marketed product), while rows E, F, and G were used for the analysis of the biosimilar mAb or mAb under various treatment. For reporting antibody, a polyclonal anti-human IgG antibody (developed by Array Bridge Inc.) was used which detected the capturing antibody-mAb complex. The reporting antibody was labeled with biotin which in turn forms a complex with streptavidin-HRP conjugate, TMB (3,3′, 5,5′-tetramethylbenzidine) was used as substrate for the HRP enzyme activity assay. Following a short development time to allow color formation from the HRP enzymatic activity, an equal volume of 1 M sulfuric acid was added to stop the reaction. A spectrophotometric plate reader (SpectraMax M3, Molecular Devices, Sunnyvale CA) was used to measure the color change at 450 nm.

## Results and discussions

### Antibody specificity

**Figure 1:**
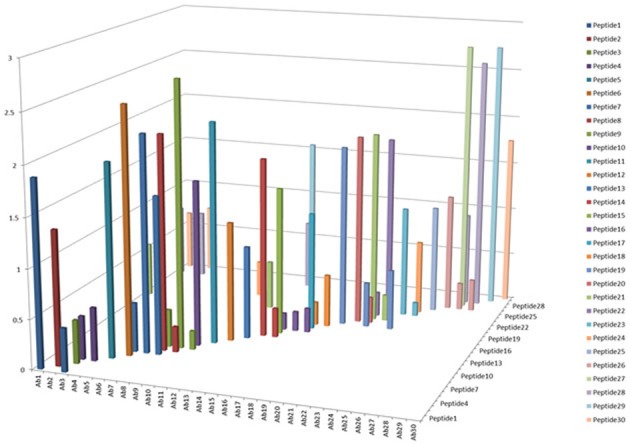
**Specificity testing of the array antibodies**. 30 antibodies generated against 30 different peptides derived from monoclonal antibody on the market were tested for their specificity in Direct ELISA. Each peptide was coated onto a 96-well plate, after blocking with 1% BSA in PBS-T (phosphate-buffered saline with 0.1% Tween-20), all the 30 different antibodies were tested against each peptide, and a total of 900 combinations will be produced. The peptide-antibody complex was further recognized by goat anti-rabbit IgG-HRP conjugate, and TMB was used as HRP substrate for color development, triplicate assays were used for the analysis.

One of the criteria required to develop a successful antibody array is the capability to probe regional changes in biologics molecules. To achieve this goal, the antibody specificity is critical. In Figure 1, each of the 30 different anti-peptide antibodies were probed with each of the corresponding 30 peptides in a pairwise analysis, i.e., each peptide was analyzed by all the 30 different antibodies and each antibody was probed with 30 different peptides. The results showed that good antibody specificity was achieved. There were a few cases of cross-reactivity from the 30 peptide and 30 antibody cross-testing (900 data points), but the overall specificity was good, suggesting that these peptide-derived antibodies could be used to probe regional changes in the mAb structure. In this pairwise testing, the main cross-reactivity occurred on the two side of the diagonal line; this was actually expected from the special design of the antibody array. In the antibody array design, peptides covering the entire monoclonal antibody light chain and heavy chain were synthesized with overlapping regions, therefore, each peptide will have two overlapping regions from its N-terminal and C-terminal ends, respectively, only the peptide corresponding to the very ends of the mAb light chain and heavy chain will have one overlapping region. Because of this special design, the polyclonal antibodies generated from one peptide could potentially recognize the adjacent peptides, and for the same reason, the antibodies generated from the two adjacent peptides could also recognize the peptide in the middle.

### Sensitivity of the assay

**Figure 2:**
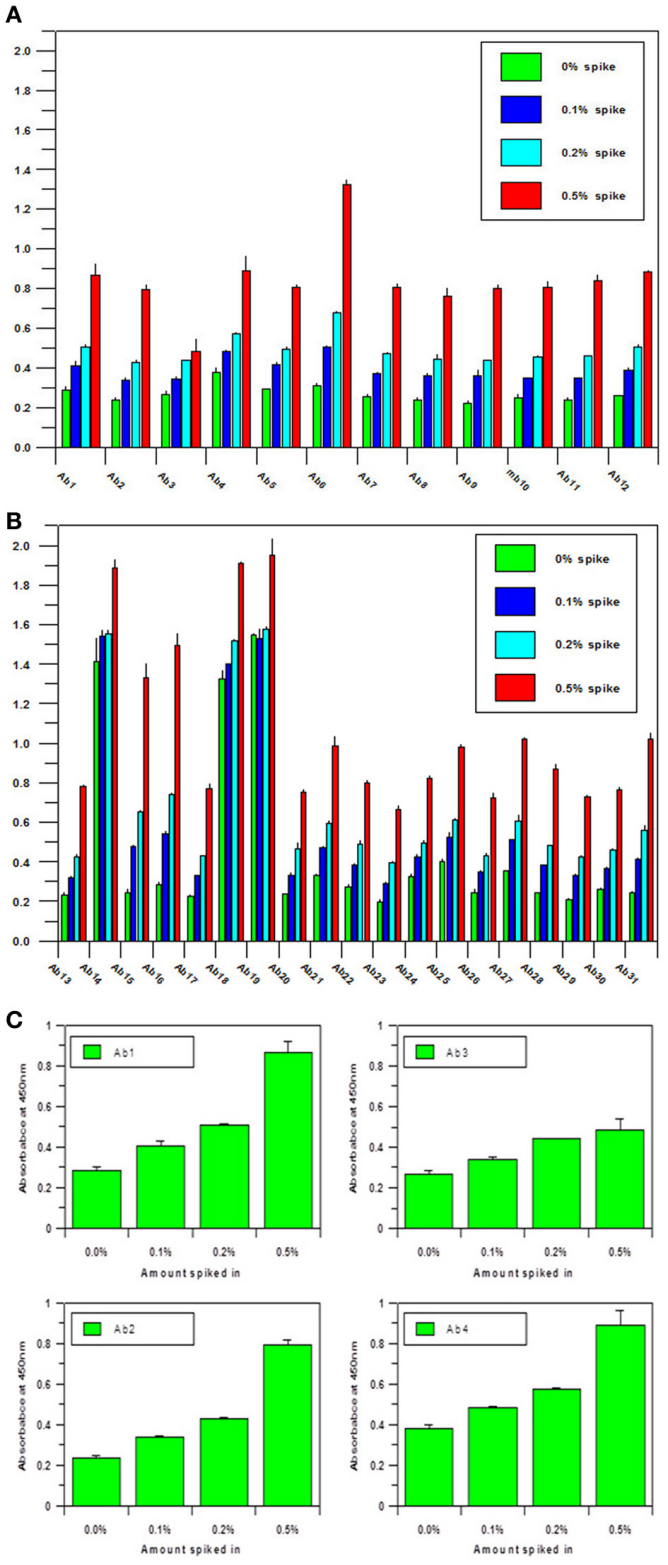
**(A)** Sensitivity of the antibody array ELISA for mAb surface epitope exposure in the variable region.A monoclonal antibody under clinical development was treated with 8 M urea and spiked into the same native mAb at 0, 0.1, 0.2, and 0.5%, respectively. Sandwich ELISA was used to measure the surface epitope exposure of each mAb samples. A final mAb concentration of 5 μg/ml was used for the analysis. **(B)** Sensitivity of the antibody array ELISA for mAb surface epitope exposure in the constant region. Same conditions as in **(A)** were used for the testing except the antibodies tested were against the constant regions of the mAb. **(C)** Quantitation of Epitope Exposure using the Antibody Array ELISA Standard Curve. For the quantitative measurement of surface epitope exposure in different regions of the mAb, a standard curve could be generated with the different levels of unfolded mAb induced from 8 m urea treatment.

Another important parameter for the antibody array technology is the sensitivity of the assay. As a useful analytical technology, it needs to detect either regional changes of the whole mAb population or changes in a sub-population which could be closely related to the mAb's efficacy and safety. To estimate the sensitivity of the technology, an artificial sample with unfolded mAb molecule needs to be generated. In the sensitivity testing, unfolded mAb was induced by 8 M urea and spiked into native mAb at 0, 0.1, 0.2, and 0.5%, respectively; it was demonstrated that as low as 0.1% spike will result in a significant increase of ELISA reading, suggesting that at least 0.1% novel epitope exposure could be detected and quantified by the antibody array (Figures 2A,B). Compared with other analytical methods used for mAb HOS analysis, antibody array technology is a more sensitive method. More importantly, the antibody array method is an accurate analytical method with variation typically less than 15%. Figure 2C is an example of building a standard curve of epitope exposure using 8 M urea-treated mAb, as shown here, for each region of the mAb, a standard curve could be built to accurately measure the epitope exposure.

**Figure 3:**
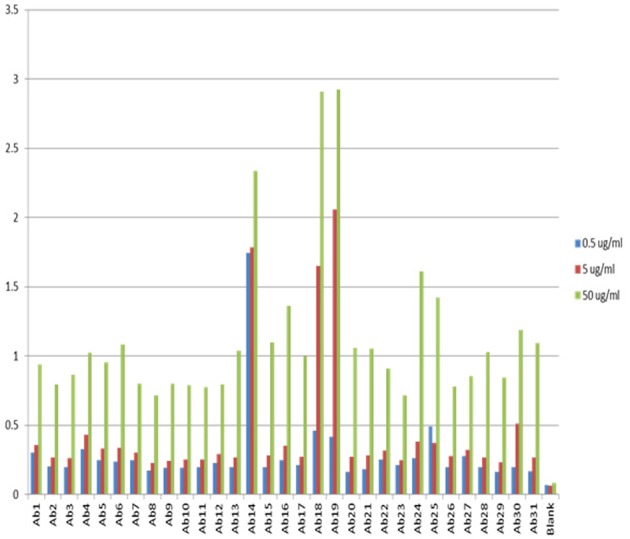
**Sensitivity of the antibody array ELISA at different mAb concentrations**. The sensitivity of the antibody array ELISA could be adjusted from the concentration of mAb used. If the intension is to detect an even smaller population of mAb with defined epitope exposure, a higher mAb concentration could be used such as 50 μg/ml. On the other hand, if it is a preliminary testing of surface epitope exposure, a low mAb concentration could be used such as 0.5 μg/ml. The standard recommended mAb concentration for testing is 5 μg/ml.

One of the advantages for antibody array technology is that the sensitivity of the method can also be increased with increased mAb concentration. Figure 3 show the assay results with mAb concentration at 0.5, 5, and 50 μg/ml, respectively. As can be seen, the sensitivity of the ELISA increases in proportion to the concentration of the testing mAb. For a typical assay, 5 μg/ml mAb is recommended. It is important to point out that because of the large size of monoclonal antibodies and the complex structure of the molecule, many of the linear epitopes the antibody array raised against are not exposed on the surface of the molecule, therefore, at any time, the antibody array can only detect a sub-population of the mAb for which the corresponding epitopes are exposed on the surface. This sub-population can be considered as a “conformational impurity” if the majority of the mAb has the epitopes buried inside. It is believed that this measurement of the “conformational impurity” will provide interesting information on how comparable the biosimilar mAbs vs. innovator molecules or how two batches of the same mAb match up conformationally in change control during novel mAb development. It is also believed that this “conformational impurity” may also be related to the mAb immunogenicity potential and/or its efficacy. For example, in the biosimilar mAb development, one can assume that corresponding to each region of the mAb; the innovator molecule has a sub-population of molecules with epitopes exposed on the surface. This sub-population can be considered as clinically tolerant “conformational impurity.” During the development of biosimilar molecules, the more epitopes exposed from this region, the more “conformational impurity” will exist. Throughout the whole molecule, if the “conformational impurity” breaks an immunological tolerant threshold, immunogenicity may occur. Currently it is not possible to quantify the relative risk these “conformational impurities” will bring to the biosimilar molecule, but one can infer that the more epitopes exposed in the biosimilar molecule as compared to the corresponding innovator mAb, the more risk it will bring to the biosimilar molecule. With the capability of the antibody arrays to detect regional “conformational impurities” for any mAb under development, it is possible to implement this technology as one of the selection methods, together with bioassay, stability testing and glycosylation, to select cell line(s) that have molecular characteristics as close to the innovator mAb as possible including the “conformational impurity” profile to ensure a successful effort in biosimilar development. On the other hand, in novel mAb development, the antibody array technology can also be used for comparability studies in both process development and formulation development as well as in change controls to ensure that the mAbs produced bear similar “conformational impurity” profile.

Another point to make about this ELISA-based assay is that the signals detected may not have a linear relationship with the content of the “conformational impurity.” This is mainly due to the nature of the ELISA assay. As we know, ELISA standard curve is a sigmoidal curve, it is only in the middle of the curve that some linear relationship could be achieved; therefore, it is important to bear this in mind when interpreting the antibody array findings. Sometimes, a 25% increase in the ELISA signal may reflect a 100% increase in the “conformational impurity” as was shown by the spiking test in Figure 2 using 8 M-urea unfolded mAb.

### Stability testing

**Figure 4:**
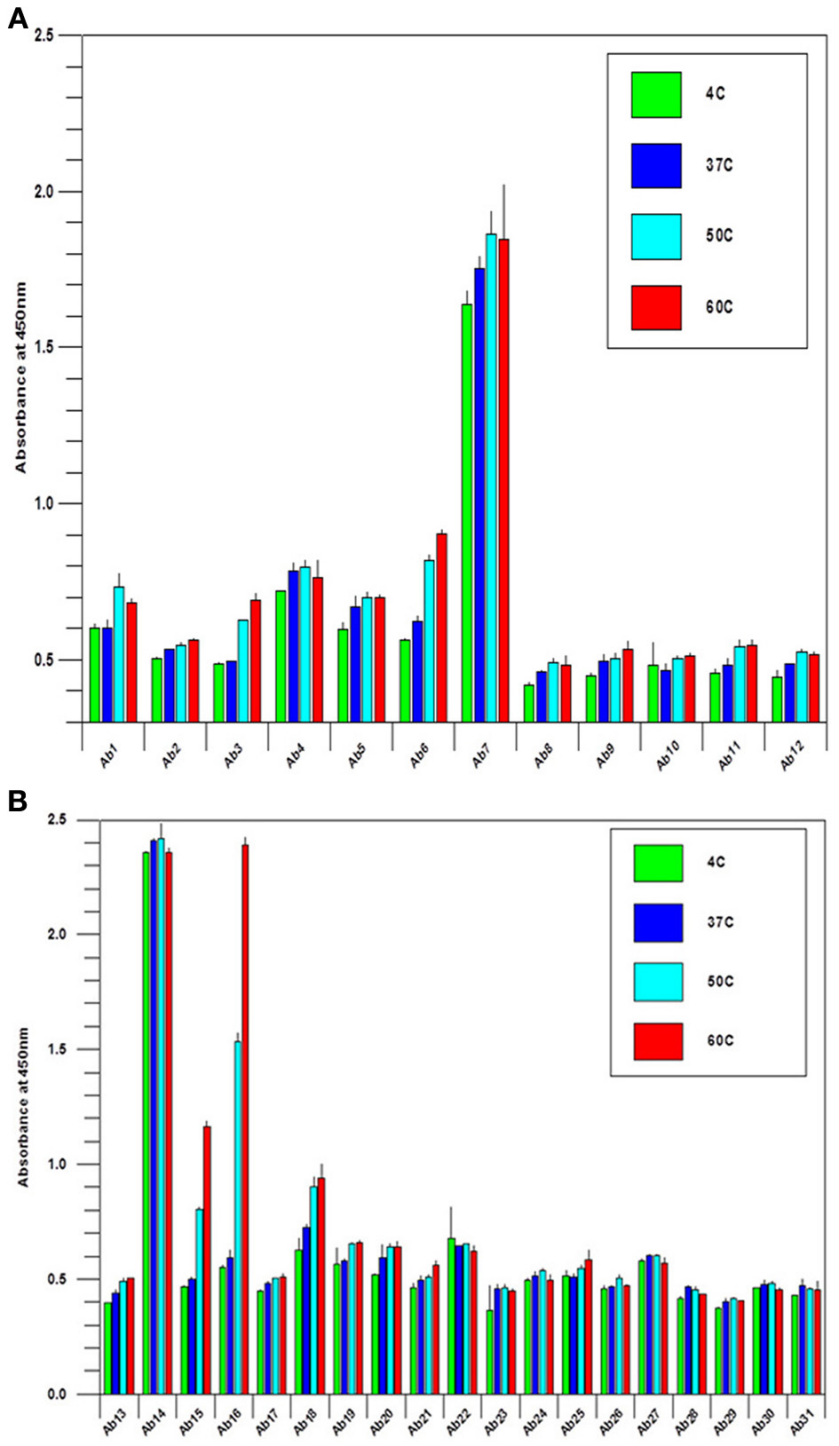
**(A)** Stability testing of mAbs under clinical development, the variable region. A mAb under clinical development was treated at different temperature as indicated overnight at 1 mg/ml, and the samples were diluted to 5 μg/ml to test the surface epitope exposure for the whole molecule. **(B)** Stability testing of mabs under clinical development, the constant region. the condition is the same as in **(A)** except the testing are for the mAb constant regions.

During biologics development, molecular stability is a major concern because of the many known pathways to protein degradation. Examples of protein degradation pathways that may lead to the instability of the biologics under development are protein deamidation, oxidation, fragmentation and disulfide bond mis-formation, therefore, formulation development to stabilize the protein is one of the most important aspects of biologics development. Here the “conformational impurity” that is measured with antibody array can also be considered as a form of “protein degradation” at higher structural level. Another important application for antibody array technology is in mAb stability testing. Figures 4A,B shows the analysis of a mAb under clinical development that was treated under different temperatures. As can be seen from the figures, different regions of the mAb responded to temperature differently. Some of the more sensitive regions of the mAb include CDR2 of the light chain, the joint region of the light chain Fv and Fc domains, and more drastically in the mAb hinge region. Previous studies have demonstrated that the mAb hinge region structure is closely related to its biological function (Klein et al., 1981).

### Applications in biosimilar mAb development

For the development of biosimilar mAbs, comparability analysis for biosimilarity is a major focus. Various analytical technologies and bioassays are needed to demonstrate comparability from many aspects of the molecule including primary and secondary structure, post-translational modifications especially glycosylation, bioactivity and protein (HOS). For protein HOS, current technologies can only provide an average reading of the molecular changes in the mAb population, changes in the specific regions of the mAb cannot be obtained. In a recent publication from the FDA, the regulatory agency acknowledges the need to develop novel technologies for protein conformational analysis (US Food and Drug Administration, 2012). It is understood that mAb Higher Order Structural changes in biosimilar mAbs could raise the potential risk of immunogenicity or altered PK/PD thus, impacting the safety and efficacy of the molecule. The antibody array technology described in this report could provide the sensitivity to detect regional changes for as low as 0.1% of new epitope exposure and provide a systematic coverage for the whole molecule. Since this is an ELISA-based assay, changes in specific regions can also be quantified. In summary, this technology could provide detailed information for the evaluation of biosimilar mAb conformational comparability.

**Figure 5:**
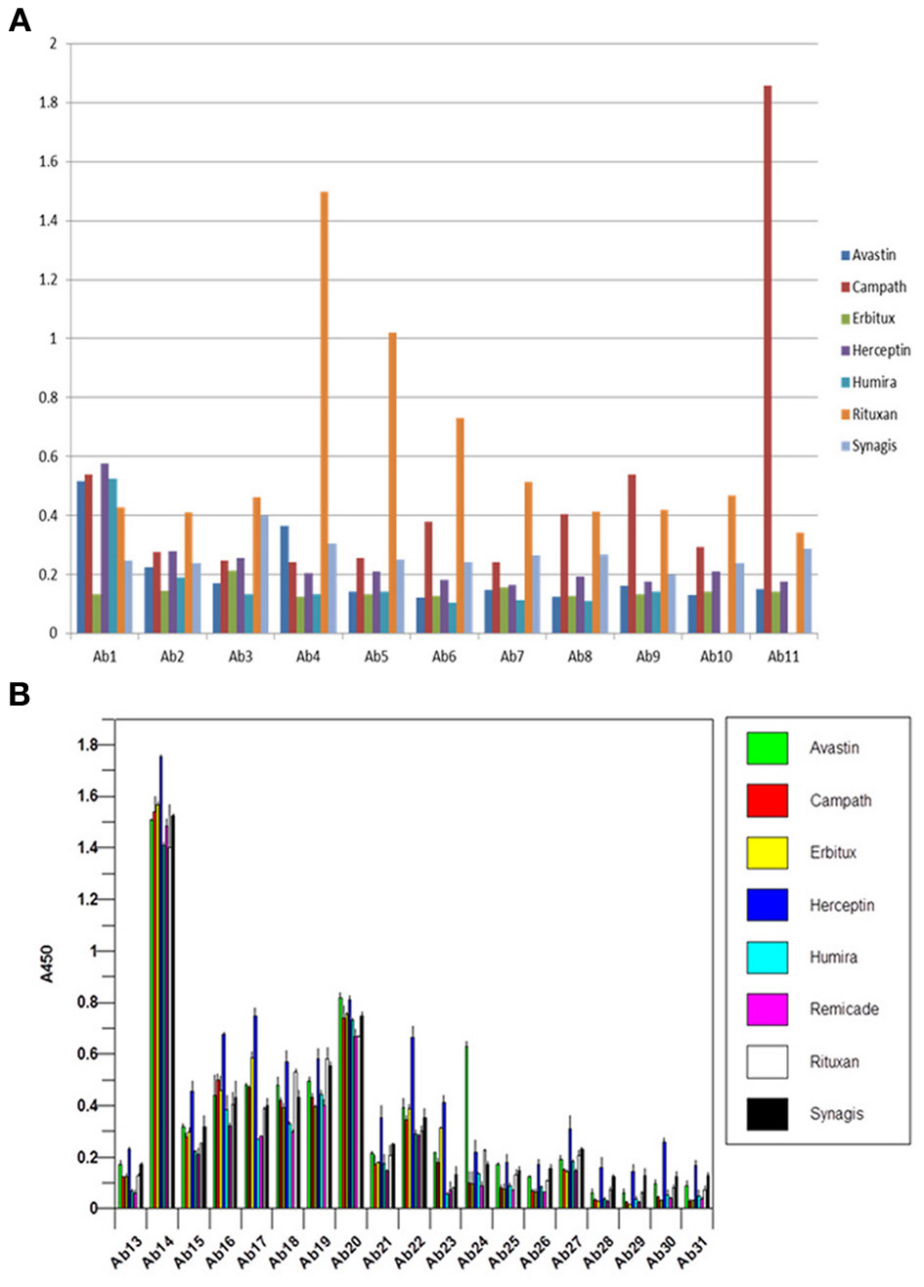
**(A)** Antibody array ELISA analysis of seven marketed mAbs, variable region. Seven mAbs on the market were tested for their surface epitope exposure using specific antibody sets prepared against the corresponding mAb variable region. **(B)** Antibody array ELISA analysis of seven marketed mAbs, constant region. Because all the mAbs on the market have highly homologous constant region with more than 95% amino acid identity, a single set of antibody raised against Herceptin constant region was used for the analysis of surface epitope exposure of all the seven mAbs on the market.

For currently marketed mAbs, the constant regions display greater than 95% sequence identity and thus, are difficult to distinguish by analytical methods (Wang et al., 2009). One of the interesting observations in the analysis of reference standards for biosimilar mAbs is that when seven of the marketed mAbs were analyzed using the antibody array ELISA, each mAb displayed a unique and different profile (Figures 5A,B), indicating that there are sequence-independent factors that play an important role in the mAb HOS. There could be many factors contributing to the unique profile in the constant region for each of the mAbs tested. One factor is the different CHO cell lines used for the production of different mAbs. It is known that different clones of the same gene in the same cell line could produce mAbs with very different properties such as molecular stability, glycosylation patterns etc. Other likely factors include differences in mAb purification and formulation.

**Figure 6:**
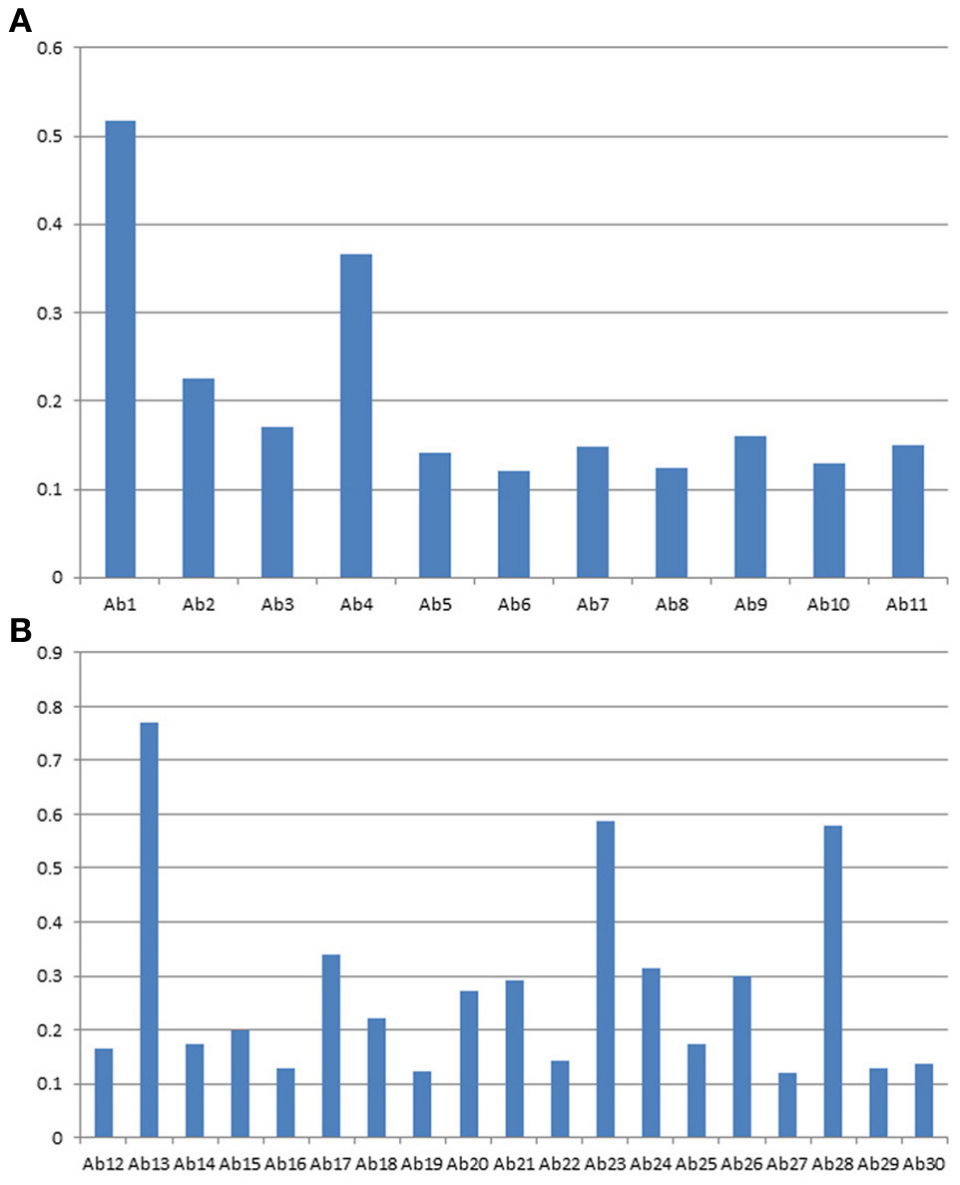
**(A)** Avastin variable region profile analyzed with antibody array ELISA. A sequence-specific set of antibodies against Avastin variable region were used for the analysis of surface epitope exposure. **(B)** Avastin constant region profile analyzed with antibody array ELISA. The common set of antibodies designed against the Herceptin constant region were used for the analysis of surface epitope exposure.

Figures 6A,B are a close up analysis of one of the most widely developed biosimilar candidates, Avastin. As can be seen, different regions of the molecule respond to the antibody array differently, suggesting different epitope distribution from these regions. However, it is important to point out that for multiple batches of the same mAb, most of the regions respond to the antibody array similarly, indicating similar epitope exposure population from batch to batch. On the other hand, we have also seen a few regions that showed batch variation for certain mAbs on the market (data not shown).

### Biosimilar mAbs conformational comparability

The antibody array technology described in this report has been used by many of the major biosimilar mAb developers in the world and assay results that are available could be categorized into three groups. In the first group minor conformational differences between the innovator molecule and the biosimilar candidates were detected, the majority of the biosimilar mAbs tested fell into this group. In this group, usually just one or two regions of the biosimilar mAb show differences compared with the innovator molecule, and the differences are typically equal to 0.1% novel epitope exposure or less as estimated with the urea-treated unfolded mAb. In the second group, there was no recognizable difference between the innovator mAb and the biosimilar molecule. Since the relative variation of the ELISA is typically less than 15%, this means that in all the regions tested, no more than 15% difference was observed. So far only a few biosimilar mAbs tested belong to this group, underscoring the challenge to produce structurally high similar mAbs in biosimilar development. In the third group, significant differences between the biosimilar molecule and the innovator mAb were detected. A few biosimilar mAbs were found to be in this group. In some cases where significant differences were observed in the HOS, the corresponding bioassays also detected loss of activity in stability testing (data not shown). While it is difficult to predict the clinical consequences of the biosimilar mAbs with the minor conformational differences, it is obvious that the biosimilar mAbs with significant conformational and bioassay differences cannot be developed clinically. It is important to point out that while in some cases the bioassay results were correlated with the conformational findings, in other cases, no bioassay differences were observed yet significant differences were observed by the antibody array testing. This is understandable because bioassays are mainly measuring changes in the CDR regions or the Fv domains, whereas the antibody arrays provide a systematic measurement throughout the molecule including the Fc region of the mAb, and this additional capability of the antibody array provides valuable information on the conformational comparability in the Fc region which could be important for the immunogenicity potential of the biosimilar mAb. Since the antibody array ELISA is an easy to use method, it might be worth the effort to implement this method when selecting cell lines producing the biosimilar mAb so that a molecule with HOS that is highly similar to the innovator molecule could be selected to ensure a successful clinical development. The fact that a few biosimilar mAbs under development showed highly similar HOS suggested that this is a possible approach.

### Antibody array provides detailed structural information

**Figure 7:**
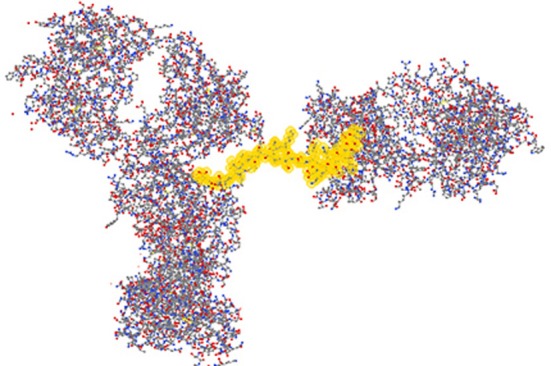
**Three-dimensional assignment of changes detected with the antibody array ELISA**. Vector NTI Express software from Life technology was used to display the mAb three-dimensional structure based on the information from the protein structure database. The fragment highlighted in yellow is one representative peptide that was used to raise the specific antibody. Epitope exposure changes detected by this antibody therefore, could be assigned to this specific region because the high specificity of the antibodies generated.

Because of the antibody specificity, changes observed by the antibody array can be assigned to a very specific region of the molecule. In Figure 7, the 3-D structure showed one region that has been detected with differences in the mAb stability testing. With this structural information for biosimilars, a method is available that can be used in many stages of development. The most valuable application will be in the cell line development because it is possible to screen multiple clones and select the one that is most close to the innovator molecule in many aspects of the attributes including its HOS. Since the mAb purification process and formulation can also impact the specific HOS the mAb will assume, this ELISA-based conformational analysis can also be used in process and formulation development.

### Novel mAb higher order structure analysis

**Figure 8:**
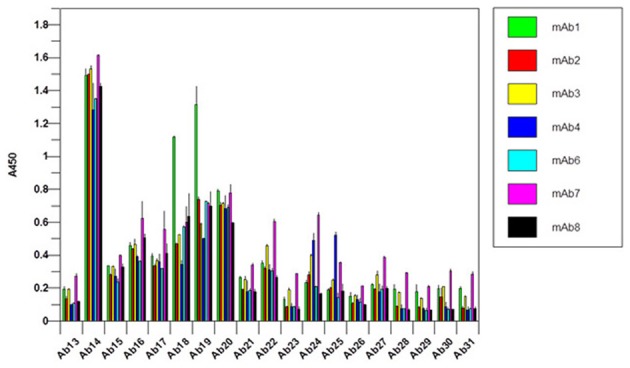
**Constant region analysis of novel mAbs under clinical development**. Seven mAbs under clinical development were tested for their surface epitope exposure, the common set of antibodies raised against Herceptin constant region was used for the testing.

It is obvious that there is a difficulty in the development of an antibody array for novel mAbs conformational analysis because each mAb will have different CDR regions. For the constant regions of light chain and heavy chain, high amino acid sequence homology exists (>95%), therefore, one set of polyclonal antibodies in these two regions will provide a good coverage for any novel mAbs (Wang et al., 2009). For the Fv regions, a platform-based approach seems count-intuitive. However, when multiple mAb sequences were aligned and compared, it is obvious that even in the Fv regions, about 2/3 of the amino acid sequence is conserved (Wang et al., 2009). The major differences only occur in the three CDR regions of the light chain and heavy chain, respectively. Furthermore, even in the CDR regions, there is also certain level of homology; therefore, the sequence analysis provides the basis for an approach for antibody array-based novel mAb HOS analysis. For the constant region of a novel mAb (2/3 of the whole mAb), a platform-based approach was taken. For the Fv region, in each overlapping peptide segment (for example amino acid sequence 1–30), the polyclonal antibodies corresponding to 8 different mAbs with known sequences were combined to provide coverage for the possible variations in a novel mAb (for example the first antibody for the variable region of the light chain of Avastin, Rituxan, Herceptin, Humira, Erbitux etc. will be combined). The reasoning here is that even if not matched exactly for a novel mAb, the polyclonal antibodies generated from the eight different mAbs will provide a good approximate for a novel mAb and actual testing indicated that this is indeed the case. In Figure 8, the constant regions of 7 novel mAbs under clinical development were tested, among them mAb3, mAb4, and mAb7 were known to have failed during the clinic. It seems that for the failed mAb, significant C-terminal epitope exposures were observed (as indicated by the red bar). Since this is a small sample pool, it is difficult to gauge the predictive value of this antibody array for novel mAb clinical outcome but it is an area that future effort will be focused on.

## Conclusions

The antibody array ELISA has been used for the conformational analysis of biosimilar as well as novel mAbs under development. The findings in biosimilar mAbs suggested that this antibody array technology can detect “conformational impurities” that are correlated with bioassay or stability findings. More importantly, this antibody array technology can detect conformational differences that sometimes may not be able to be detected with other current analytical technologies or bioassays, suggesting a complementary value in biosimilar as well as novel mAb development. It is possible to implement this assay in biosimilar mAb cell line development to ensure that highly similar mAbs with conformational comparability could be selected. In novel mAb development, it was shown that the InnoBridge ELISA kit could be used to measure conformational comparability of mAbs under clinical development. Furthermore, a small sample of mAbs that were known to have failed during clinical development seem to have more C-terminal epitope exposure compared with those that have made it to the market. Finally in the mAb constant region, whether it is mAbs on the market or novel mAbs under development, no two mAbs gave identical conformational pattern even though the amino acid sequences are almost identical. This underscores a point that is taken as fact in the biosimilar field, that “process is the product.” It also indicates the challenge to develop biosimilar mAbs because the detailed process information from the innovator companies is not known for the most part. As more sensitive and accurate analytical technologies for mAb characterization are developed and implemented, the development of biosimilar as well as novel mAbs will be put on a solid scientific base.

### Conflict of interest statement

The authors declare that the research was conducted in the absence of any commercial or financial relationships that could be construed as a potential conflict of interest.
